# Increasing the Versatility of Durum Wheat through Modifications of Protein and Starch Composition and Grain Hardness

**DOI:** 10.3390/foods11111532

**Published:** 2022-05-24

**Authors:** Domenico Lafiandra, Francesco Sestili, Mike Sissons, Alecia Kiszonas, Craig F. Morris

**Affiliations:** 1 Department of Agriculture and Forest Sciences, University of Tuscia, 01100 Viterbo, Italy; francescosestili@unitus.it; 2 NSW Department of Primary Industries, Tamworth 2340, Australia; 3 United States Department of Agriculture, Agriculture Research Service, Western Wheat Quality Lab, Pullman, WA 99164, USA; alecia.kiszonas@usda.gov (A.K.); craig.morris@usda.gov (C.F.M.)

**Keywords:** durum wheat, grain hardness, D-genome glutenin subunits, dough strength, waxy, amylose

## Abstract

Although durum wheat (*Triticum durum* L. ssp. *durum* Desf.) has traditionally been used to make a range of food products, its use has been restricted due to the absence of the D-genome glutenin proteins, the relatively low variability in starch composition, and its very hard grain texture. This review focuses on the manipulation of the starch and protein composition and modification of the hardness of durum wheat in order to improve its technological and nutritional value and expand its utilization for application to a wider number of end products. Starch is composed of amylopectin and amylose in a 3:1 ratio, and their manipulation has been explored for achieving starch with modified composition. In particular, silencing of the genes involved in amylose and amylopectin synthesis has made it possible to isolate durum wheat lines with amylose content varying from 2–3% up to 75%. This has created opportunities for new products with different properties and enhanced nutritional value. Durum-made bread has generally inferior quality to bread made from common wheat. Attempts to introduce the *Glu-D1* subunits 1Dx5 + 1Dy10 and 1Dx2 + 1Dy12 produced stronger dough, but the former produced excessively strong, inelastic doughs, and loaf volume was either inferior or not affected. In contrast, the 1Dx2 + 1Dy12 sometimes improved bread loaf volume (LV) depending on the glutenin subunit background of the genotype receiving these genes. Further breeding and selection are needed to improve the dough extensibility to allow higher LV and better texture. The versatility of durum wheat has been greatly expanded with the creation of soft-textured durum via non-GMO introgression means. This soft durum mills like soft hexaploid wheat and has similar baking properties. The pasta quality is also not diminished by the soft-textured kernels. The *Glu-D1* locus containing the subunits 1Dx2 + 1Dy12 has also been introgressed to create higher quality soft durum bread.

## 1. Introduction

Durum wheat (*Triticum turgidum* subsp. *durum*) is widely grown in the Mediterranean area representing a major staple crop used for the preparation of different end products whose history and utilization have accompanied the journey of man for thousands of years. Though commonly known for its use in pasta, durum wheat, also thanks to its glassy texture, is used for the preparation of different types of bread, biscuits, pastries, and other kinds of foods, and the processing can include whole or crushed kernels [1]. About 25% of the durum wheat produced in the world is used for breadmaking and up to 70–90% in some Middle East countries [2,3]. This review covers approaches used to manipulate the (1) protein composition to improve durum breadmaking quality, (2) starch composition to modify the amylose/amylopectin ratio to alter the nutritional value, and (3) durum wheat kernel hardness to a soft texture for expanded use of durum by making milling accessible to those with traditional roller mills without sacrificing pasta quality.

It is generally known that durum makes pan breads that are inferior in their bread-baking performance to that made from hexaploid wheat varieties. Specifically, durum bread has inferior loaf volume, structure, and texture compared with common wheat bread [4,5,6,7,8], although durum bread is preferred in some markets for its peculiar and distinctive sensory properties, with a better shelf life than conventional bread [2]. However, it is possible to produce acceptable bread made from blends of durum with bread wheat flour [9].

Although increasing protein content can improve loaf volume, the typically inelastic and poorly extensible gluten in durum prevents full gas expansion, as dough extensibility is an important trait for obtaining good loaf volume [4]. Guzman et al. [7] noted that several durum varieties produced loaf volume similar to bread wheat in some environments, particularly under drought stress, indicating the potential for durum in breadmaking.

The inelastic nature of durum gluten is related to the glutenin subunit composition. Two factors are important: the total number of high molecular weight glutenin subunits (HMW-GS) and the absence of the HMW-GS associated to the D genome. The maximum possible number of HMW-GS in durum wheat is three, compared with the hexaploid wheat maximum of five [10]. The HMW-GS enables the strong doughs that are critical in baking for trapping small bubbles of carbon dioxide gas formed by yeast during proofing, thereby enabling the dough to rise and ensure a good loaf volume and structure to leavened bread. In bread wheat, the most prominent locus that contributes to dough elasticity and extensibility is the *Glu-D1* locus, with two allelic variants, 1Dx2 + 1Dy12 (2 + 12) and 1Dx5 + 1Dy10 (5 + 10). Functionality studies of specific HMW subunits have found that subunit 1Dx5 + 1Dy10 is considered the “stronger” allele and is more desirable for bread quality prepared from hexaploid wheat [11,12]. Durum wheat lacks the D genome, and thus, it also lacks the *Glu-D1* locus. Consequently, the elasticity and extensibility of durum doughs are often viewed as inferior to bread wheat [4]. Dough strength results from a complex interplay between the HMW glutenin subunits, low molecular weight glutenin subunits (LMW-GS), gliadins, and non-protein endosperm constituents. Transferring the storage protein genes that are present on chromosome 1D into durum has been attempted to try to improve durum breadmaking quality.

A technology that has been very effective in introducing D-genome-related proteins into durum wheat is chromosome engineering. In wheat normally, recombination is restricted to homologous chromosomes from the same genome due to the presence of the *Ph1* locus present on the long arm of chromosome 5B. The availability of mutants (*ph1*) with a deletion at this locus has resulted in the possibility of inducing homoeologous pairing (similar chromosomes from different genomes, e.g., 1A vs. 1B or 1A vs. 1D) and realize the transfer of chromosomal segments, carrying genes of interest from wild or cultivated wheat into bread and durum wheat [13].

However, for good pasta quality, it has been shown that the most important genes are those associated at the *Glu-A3*, *Glu-B3*, and *Glu-B2* loci, which encode the B-type LMW-GS [14,15,16]. Broadening the uses of durum wheat to improve breadmaking quality while maintaining pasta-making quality is a desirable goal.

Starch makes up more than 75% of the wheat kernel and represents an important source of energy in the human diet, contributing to >50% of caloric intake in the Western world and up to 90% in developing countries [17]. According to Hardy et al. [18], starch has played an essential role in human evolution by providing accessible carbohydrates to promote a significant increase in brain size. Starch contributes greatly to the textural properties of many foods and is widely used in industrial and food applications as a thickener, colloidal stabilizer, gelling agent, bulking agent, and water retention agent [19]. Starch is composed of two different types of polymers, amylopectin and amylose, in a 3:1 ratio, differing in degree of polymerization and number of side branches. Amylose is essentially formed by a linear chain of D-glucose molecules with a low degree of polymerization, whereas amylopectin shows a higher degree of polymerization. Together, the two polymers are assembled to form insoluble semi-crystalline starch granules.

The application of mutagenesis in association with reverse genetics has provided a new powerful advance in both functional genomics and crop-breeding applications: A combination of chemical mutagens with PCR detection of point mutations in a gene of interest for which the sequence is known has resulted in the development of the TILLING (Targeting induced local lesions in genomes) strategy [20]. Chemical mutagens such as ethyl methane sulfonate (EMS) are very effective in inducing randomly distributed single-nucleotide changes in genomes of many different crop species. Its use generates allelic series of mutations, including missense changes, with amino acid substitutions that can have a range of effects on protein function and non-sense or splice site changes that can cause truncation of the gene product and, depending on the location, probable loss of function. Such an approach has been used to generate and detect mutations in different types of genes including those involved in the biosynthesis of amylose and amylopectin in durum and bread wheat [21,22,23,24,25].

Durum wheat traditionally has a very hard and glassy-textured kernel. This hard texture necessitates specialized durum mills, as opposed to mills for hexaploid wheat, which can be changed easily for hard and soft bread wheat. Not only are durum mills specialized, but they produce semolina. Semolina is a much coarser product with a greater average particle size (~250–300 µm). Durum flour can be made with a particle size distribution more similar to that of bread wheat (~80–100 µm), but the level of damaged starch is so great that the flour is challenging to work with as a base flour [26,27]. The particle size and properties of semolina make durum best for pasta and couscous but limit its further uses. In bread, wheat grain hardness is an important parameter that influences its mechanical properties, milling behavior, flour yield, starch damage, water absorption, dough rheological properties, volume, and crumb structure [28]. Grain hardness is primarily controlled by the genes present at the Hardness locus, *Ha*, present on the distal part of the short arm of chromosome 5D, which encodes the so-called Puroindolines a and b [28,29]. Both genes, when present in their wild-type (*Pina-D1a* and *Pinb-D1a*), create a soft-textured kernel. Any changes in either gene create a harder texture [30]. Traditionally, soft-textured wheat is used for cookies, cakes, pastries, etc. whereas hard wheat is used for bread. More recently, to increase the versatility of durum, both of the wild-type alleles of the puroindoline genes were transferred into durum using the *Ph1* gene as described above. The result was that a small section of the distal tip of chromosome 5DS was transferred to chromosome 5BS of the durum wheat. This generated drastic changes in the milling characteristics of the grain and end-product properties [31,32]. Gazza et al. [31] introduced the two *Pina-D1a* and *Pinb-D1a* alleles in the Italian durum wheat variety Colosseo and showed that the hardness of the grain was strongly reduced as demonstrated by the low SKCS-HI values. In addition, the reduction of the kernel hardness produced several changes in the flour, such as higher flour extraction rates (24%) compared with their hard durum sister line, decreased farinograph water absorption, decreased dough tenacity (P), and, accordingly, alveograph P/L ratio, but increased farinograph stability, mixing tolerance and dough extensibility (L). Moreover, spaghetti cooking quality, as determined by firmness, stickiness, and bulkiness, was unaffected by the kernel hardness, whereas the loaf volume exhibited a 10% increase associated with kernel softening.

## 2. Introduction of D Genome Gluten Proteins in Durum Wheat

There are two approaches that can be used to introduce the *Glu-D1* subunits. Transgenic lines expressing additional HMW-GS genes have been reported to mostly involve adding subunits 5 + 10 to improve dough strength with the hope of improving breadmaking quality in bread and durum wheat [33,34,35,36,37]. These works showed increases in polymeric protein, mixing times, and mixing tolerance but often produced overly strong doughs. Gadaleta et al. [38] transformed four durum cultivars with 1Dx5 and 1Dy10. Higher SDS sedimentation volume and overly long mixograph peak time and very stable but inextensible dough resulted. Overexpression of subunit 1Dx5 in the durum wheat cultivar Ofanto led to the production of doughs that were too strong for conventional mixograph analysis, resulting in erroneously low mixing time and peak resistance [37]. Given that GMOs still lack widespread consumer acceptance, this approach has not proved effective.

**Figure 1:**
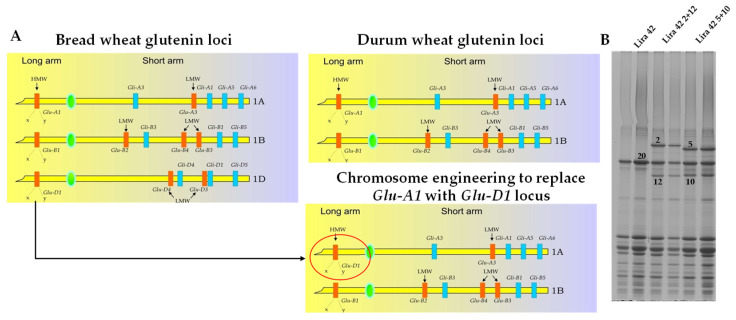
Chromosome localization of glutenin loci in bread and durum wheat. The red circle highlights the *Glu-D1* locus introgressed in durum wheat through chromosome engineering (**A**). SDS-PAGE of glutenin subunits of three durum wheats: Lira 42 and two chromosome engineered lines (Lira 42 2 + 12 and Lira 42 5 + 10) (**B**).

Using chromosome engineering, segments carrying the *Glu-D1* loci containing genes encoding the pairs 1Dx5 + 1Dy10 or 1Dx2 + 1Dy12 have been transferred in durum wheat, replacing the null allele present at the *Glu-A1* locus on the long arm of the 1A chromosome using translocation lines 1AS.1AL-1DL, in an attempt to improve durum breadmaking quality [39,40,41,42,43] (for a recent review on the effects of *Glu-D1* gene introgressions in durum wheat see Morris [44]) (Figure 1). Klindworth et al. [45] reported that dough from the durum 1AS.1AL-1DL translocation lines produced by Joppa et al. [39] had exceptionally strong mixing characteristics but did not improve loaf volume over their parental cultivars. Klindworth et al. [40] noted that RugbyT genotypes, carrying the low molecular weight glutenin subunits LMW-1 banding pattern, which conditions weak gluten, had a lower glutenin-to-gliadin ratio and better loaf volume than genotypes carrying LMW-2, associated with superior quality, suggesting that higher gliadin content and improved extensibility are needed to improve breadmaking quality of 1AS.1AL-1DL translocation genotypes.

Ammar et al. [46] backcrossed lines carrying 1Dx5 + 1Dy10, produced by Lukaszewski [41] into three durum cultivars and observed a greatly increased SDS sedimentation volume. One of these lines was crossed into the Italian cultivar Svevo, and this increased mixograph peak development time (one indicator of dough strength) from 5.1 to 15.0 min [47]. Sissons et al. [42] used these Lukaszewski [41] lines to develop BC6 NILs possessing 1Dx2 + 1Dy12 or 1Dx5 + 1Dy10 in the durum cv. Svevo, which has the *Glu-B1* 7 + 8 subunit pair present. Mixograph peak development times followed this order, Svevo < Svevo 1Dx2 + 1Dy12 < Svevo 1Dx5 + 1Dy10, the same order as that for Farinograms run at 180 rpm. The addition of 5 + 10 made the dough overstrong and inextensible, and both subunit pairs failed to improve loaf volume over Svevo. The inclusion of these subunits led to a greater amount of the larger polymeric glutenin (higher UPP%), probably due to additional disulfide bond formation, leading to dough that shows more resistance to mixing with higher dough strength. An increased glutenin-to-gliadin ratio has also been reported in lines having a 5 + 10 inclusion [40,46]. Further work by Sissons et al. [48] explored the impact of *Glu-D1* subunits on bread quality in different glutenin subunit backgrounds to see if this provided the dough with more extensibility to improve loaf volume. Durum wheat variety Svevo missing *Glu-B1* subunits 7 + 8 and Lira biotypes with low molecular weight glutenin subunit types 1 (Lira 42) and 2 (Lira 45) and HMW-GS 20, which is known for its negative effect on dough characteristics, were evaluated for their dough strength and breadmaking potential with and without high molecular weight glutenin subunits 2 + 12 or 5 + 10. The absence of subunit pair 7 + 8 in Svevo reduced the overly strong dough-strengthening effect from 5 + 10 but also 2 + 12 compared to what was found previously when 7 + 8 is present. The weak gluten variety Lira 42 genotype had stronger dough from the addition of 2 + 12 and 5 + 10, and both Lira biotypes showed much larger effects on dough strength from the *Glu-D1* pairs than with Svevo, which has moderate strength. There were minor impacts on pasta quality with 2 + 12 or 5 + 10 additions, which should allow flexibility to develop durum with a better balance of glutenin subunits more suited to bread making [49].

Bread prepared using blends of durum flour with commercial wheat flour indicated that at any blend level, durum flour reduced loaf volume in a dose-dependent manner, showing that none of the genotypes could match 100% hexaploid commercial flour for LV and texture. In weaker gluten genotypes (Lira 42, Lira 45) with added 2 + 12 or 5 + 10, LV improved beyond its control genotype but was inferior to 100% bread wheat loaves. However, in the stronger genotype, Svevo, there was no improvement in LV with 2 + 12 and indeed a decline in LV with 5 + 10 present [48]. This indicates that there is an interaction between the added *Glu-D1* subunits and the background glutenin composition. Key *Glu-D1* subunits critical for good breadmaking in hexaploid wheat appear to have limited value in improving loaf structure and volume in durum bread, especially when the proportion of durum to bread wheat flour increases above 25%. The balance of glutenin to gliadin subunits is still not ideal because what is needed is more extensibility in the dough. Klindworth et al. [40] determined that a translocation line carrying the LMW-1 banding pattern, which conditions weak gluten, had better loaf volume and mixing characteristics than lines carrying LMW-2, which conditions strong gluten, supporting this hypothesis. However, Ammar et al. [4] noted that durum carrying HMW-GS 6 + 8 produced bread loaves with higher LV than those produced by genotypes having 7 + 8 or 20, probably due to their higher dough extensibility. These researchers concluded that in order to produce durum wheat with baking performance equivalent to bread wheat, greater dough strength but, more importantly, extensibility is needed. The 1AS.1AL-1DL translocation lines have been reported to produce dough with very low extensibility [50]. The effect of the 2 + 12 addition does depend on the genetic background because when present in a soft durum, dough strength and bread LV were greatly improved [51] but not in lines where the 5 + 10 subunit pair was added because the dough was too strong and inelastic.

## 3. Manipulation of Starch Composition

**Figure 2:**
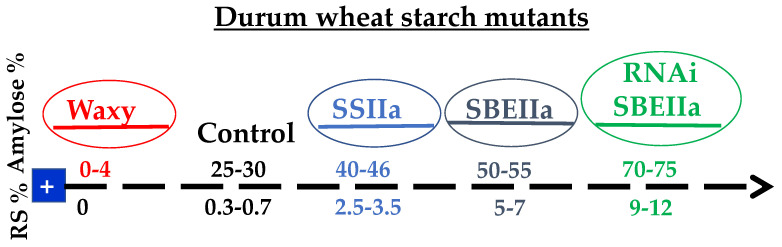
Schematic representation of amylose content and resistant starch (RS) in a panel of durum wheat starch mutants along with a transgenic line obtained through RNA interference (RNAi). SSIIa and SBEIIa indicate mutant lines not expressing the starch synthase of class IIa (SSIIa) and the starch branching enzyme of class IIa (SBEIIa).

The synthesis of amylose and amylopectin is carried out by different classes of enzymes. In particular, a granule-bound starch synthase (GBSSI) is involved in amylose synthesis, whereas amylopectin is produced by the concerted action of different starch synthases (SSI, SSII, SSIII), starch-branching enzymes (SBEI, SBEIIa and SBEIIb) and starch-debranching enzymes of isoamylase- and limit dextrinase-type (ISA and LD) [52,53,54]. The manipulation of starch composition has been the target of many researchers, thanks to the identification of mutants involved in the synthesis of the amylose and amylopectin and the availability of genomic resources and of new high-throughput technologies (Figure 2). This has made it possible to generate durum and bread wheat with large variations in amylose content and starches with unique functionalities.

### 3.1. Low Amylose Durum Wheat

The gene-encoding GBSSI enzymes, also termed waxy due to the appearance of the kernel, are located at chromosomes 7A (*Wx-A1*), 4A (*Wx-B1*) and 7D (*Wx-D1*) of bread wheat, while in durum wheat, only the *Wx-A1* and *Wx-B1* genes are present. When only one gene is silenced, partial waxy wheat is obtained, whereas when all the genes are silenced a full waxy is realised.

The different studies of these proteins have identified polymorphism at the three loci, their molecular characterisation and evaluation of their effects on starch composition [55]. The presence of one or two *GBSSI* null alleles results in the production of starch with reduced amylose content in bread wheat. Complete waxy genotypes have been produced in different durum wheat cultivars through two main strategies, the introgression of natural mutations in cultivated varieties and the use of chemical mutagenesis to suppress the activity of their encoding genes [21,56]. The first waxy wheat genotype was produced by Nakamura and colleagues [57] by crossing the bread wheat cv. Kanto 107 (a natural mutant lacking *Wx-A1* and *Wx-B1*) and the wheat landrace Baihuomai. The complete waxy genotype showed a drastic reduction of amylose (close to zero) and an endosperm with a waxy phenotype. A similar approach was followed by Urbano and colleagues [56] that produced a waxy durum wheat line by crossing the cv. Kanto 107 with the durum wheat cv. Svevo. The mutant line had low amylose content (less than 3%) [58].

The complete durum wheat waxy mutants showed drastic changes in starch composition and properties compared with the nonwaxy, suggesting possible new applications in food and non-food industries. Grant et al. [59] highlighted significant differences in the chemical and functional properties of waxy durum starch compared with the starch in the wild type. The full waxy starches had different pasting properties (by RVA) characterised by higher peak viscosities, earlier peak times, lower stabilities and final viscosities [55]. Further differences were also detected in the gelatinization properties: starches gelatinized at higher temperatures and needed more energy to be melted compared with the control durum wheat. Vignaux et al. [60] investigated the quality characteristics of the durum wheat null waxy lines, finding a slight worsening of gluten quality and semolina yields. However, the overall quality characteristics of waxy durum grain were still considered satisfactory. Complete and partial waxy durum wheat genotypes were used to evaluate the effect of waxy mutations on spaghetti quality [61]. A significant worsening was observed for all the main parameters associated with pasta quality (firmness, cooking loss, stickiness). In detail, the cooked waxy pasta was softer and had more cooking loss compared with the control pasta. The higher cooking losses were associated with the absence of the amylose-proteins interaction that is essential for forming a strong network that can trap the exudates [61].

Similarly, Gianibelli and colleagues [62] found that the incorporation of waxy starch isolated from bread wheats into semolina had negative effects on pasta quality due to the increase of stickiness and the reduction of firmness in cooked pasta. Sharma et al. [63], using partial waxy durum wheat genotypes, observed a minor impact on the worsening of pasta quality. From the above cited literature, it is evident that waxy semolina is not suitable for making pasta because of its lower firmness and increased stickiness; however, the softening properties of waxy dough may open new opportunities in other industrial applications.

More recently, Sissons et al. [48] prepared bread made from blends of a set of durum waxy flours and a commercial baker’s flour and found that the presence of the 5 + 10 improved pasta quality by increasing the firmness and reducing stickiness and cooking loss of pasta. Though durum wheat is mostly used for the production of pasta, its use for different kind of breads and pastries is very popular in the Mediterranean area, and waxy durum wheat can be used as an antistaling agent [64].

White pan bread was baked from 10, 20, and 30% waxy durum wheat flour composites and evaluated for loaf volume and crumb firmness over a period of 0, 3, and 5 days. Loaf volumes were unaffected by waxy flour blends, but as staling progressed over 3–5 days, significant firming of crumb was observed in the control sample compared with the loaves containing waxy flour. Bread firmness was inversely proportional to the level of waxy flour used in the blend, with a 20% waxy wheat flour blend being optimal in retarding staling while producing bread quality comparable with the control. It was further established that bread made with 20% waxy flour gave lower firmness values after 5 days of storage in comparison with bread made with 3% shortening [65]. According to Shevkani et al., [17] the incorporation of waxy wheat flour positively influences bread making due to the retardation of staling and the extension of shelf life. The breads prepared with waxy wheat (5–30% incorporation levels) had soft and tasty crumb and showed improved shelf life [66]. The waxy common and durum wheat flour incorporated (25%) into common flour also improved loaf expansion during baking and reduced loaf firmness of breads, attributed to the higher gelatinization temperatures and swelling ability of waxy starches that reduced overall water availability [17].

More recently, Sissons et al. [48] used the durum wheat variety Svevo (Sv), a low-amylose line (SvLA, 14,9% amylose) and SvLA 5 + 10, to make blends at 10%, 25%, 50% durum and baker’s flour and prepare 100 g loaves. Low-amylose Svevo showed similar loaf volume to Svevo, while the presence of the 5 + 10 HMW-GS had minimal impact on LV, except at 50% with a small improvement in loaf quality. Bread stored up to 7 days became firmer partly due to increased starch retrogradation, and loaves were similar to bread made from baker’s flour. Low-amylose Svevo kept the loaf fresher but only up to 3 days of storage. Subunit pair 5 + 10 made the loaf firmer after 7 days compared with control.

### 3.2. High Amylose Durum Wheat

There is strong evidence that numerous chronic health conditions could be prevented or moderated by correct dietary behaviours. In this regard, an important factor is starch digestibility. Based on the digestibility rate, the starches can be divided into rapidly digestible starches, slowly digestible starches, and resistant starches. Foods containing rapidly digested starches may contribute to the onset of chronic diseases because they are easily accessible to digestive enzymes and rapidly digested to glucose molecules with detrimental effects on human health [67]. On the contrary, several studies have demonstrated the existence of a positive correlation between the amylose content in flour or semolina and the resistant starch (RS) in foods, attracting the interest of consumers and the food industry for its role in the prevention of several diet-related diseases [68]. Indeed, RS escapes the digestion in the small intestine and reaches the large bowel, where it is fermented by the bacterial microflora, playing a role as dietary fibre. Short-chain fatty acids (SCFAs), especially butyrate, contribute to the well-being of colonocytes with a possible role in the prevention of colon cancer [69,70,71]. In addition, increases in SCFAs result in lowering of the bowel pH, and this contributes to hindering the proliferation of pathogenic bacteria and reducing inflammation in irritable bowel disease [72]. In addition, the foods with high resistant starch have low glycaemic index and can help in preventing the onset of type II diabetes and obesity [69,73]. The mechanisms responsible for the reported effects of dietary fibre on metabolic health are still being investigated, but it is speculated to be a result of changes in intestinal viscosity, nutrient absorption, rate of passage, production of short chain fatty acids and production of gut hormones [74]. The beneficial effects for human health associated with the consumption of RS have promoted a growing interest in increasing the amount of amylose in cereal grains [73].

Two main strategies have been followed to raise the amount of amylose in wheat. The first one has focused on the silencing of a key gene of amylopectin biosynthesis involved in the formation of α-1,4 linkages, corresponding to the starch synthases of class IIa (SSIIa) [58,75]. The second strategy targeted another key gene of amylopectin synthesis involved in the formation of α-1,6 linkages, corresponding to the starch branching enzyme of class IIa (SBEIIa) [21,23,24,76]. Lafiandra et al. [58] produced a durum wheat mutant line with an increased amylose content by about 89% compared with the control (37.3% vs. 28.9% amylose) by introgressing, in the cultivar Svevo, null mutations on *SSIIa-A* and *SSIIa-B* homeoalleles, previously identified in bread wheat [77]. A set of 14 SSIIa mutant lines, derived from the backcross with Svevo, was subsequently characterized by Botticella et al. [78], who investigated some major traits such as amylose, resistant starch, starch content, arabinoxylans, α-glucans, seed colour and thousand grain weight. These analyses highlighted large variability for all the parameters among the fourteen lines; the amylose content ranged from 37% to 46% and was positively correlated to the content of resistant starch, which reached up to 3.2% vs. 0.4% in Svevo, confirming their elevated nutritional value. However, the major issue of SSIIa mutant lines is the drastic reduction of some parameters associated with yield (total starch, thousand grain weight) [77].

Hogg et al. [75] produced a new set of partial and complete SSIIa mutants in durum wheat by combining natural and EMS-induced mutations. The authors introgressed a natural knockout mutation on the *SSIIa-A1* gene in the variety Montrail and treated the obtained lines with a chemical mutagen with the aim of knocking out the other homoeoallele (*SSIIa-B1*). Double null SSIIa mutant lines showed a drastic reduction of total starch (up to ~33%); the loss in seed weight was significant but less important (−6%) compared with the reduction reported by Botticella et al. [78], which ranged from −32% to −46%. Martin et al. [79] investigated the influence of the *null* allele at the locus *SSIIa-A* on rheological properties and noodle quality in two segregating durum wheat populations derived from the cross of two SSIIa-A1 null mutants (PI330546 and IG86304) with the cultivar Montrail [75]. Although the swelling power was lower in both the crosses, the amylose amount and noodle firmness were higher in the IG86304 cross compared with the control (cv Montrail), whereas no significant differences were observed in the other cross. In a more recent study, the same research group evaluated pasta quality and nutritional value of the SSIIa null durum wheat genotype compared to the wild-type control line, reporting an increase in pasta firmness and resistance to overcooking, parameters that have a positive influence from a quality point of view [80]. Nevertheless, negative quality effects were described about pasta cooking time and colour, which were diminished, and cooking loss, which increased. From a nutritional view, high amylose pasta had several improved attributes, such as an increased amount of resistant starch and dietary fibre and fewer free carbohydrates. The lower release of carbohydrates, in particular glucose, in the stomach has been associated with beneficial effects for human health in the prevention of diet-related diseases, such as type II diabetes and obesity [81].

The second strategy, based on the suppression of the activity of the two paralogs *SBEIIa* and *SBEIIb*, present in cereals, proved to be more efficient in increasing the amount of amylose in wheat. In bread wheat, Regina et al. [82] used RNA interference and showed that the silencing of the *SBEIIa* isoform was associated with a highly increased proportion of amylose in the transgenic lines (up to 70% of total starch).

Sestili et al. [24] used the RNA interference strategy to down-regulate *SBEIIa* genes in order to increase the amylose content in two durum wheat cultivars, Svevo and Ofanto. Genetic transformation was carried out with the biolistic approach for the cultivar Svevo and with Agrobacterium for Ofanto. The silencing of the *SBEIIa* gene caused marked modifications to amylose content, starch composition and granule morphology. Amylose values ranged from 30.8% up to 75% for Svevo and from 27.7% to 56.4 for Ofanto. Both grain weight and total starch were significantly decreased in all the SBEIIa null lines compared with the controls, but the reduction was lower than that observed in the SSIIa null genotypes.

Following the first attempts to modify starch composition by transgenic approaches, the introduction of the TILLING approach, a non-GM technology, was soon explored in bread and durum wheat [21,22,23,76]. The silencing of these *SBEIIa* genes by a TILLING approach increased the amylose and resistant starch up to 47.4% and 6.21% in the cultivar Kronos [21] and up to 52.7% and 6.79% in the cultivar Svevo [76], respectively. A modest increase of amylose (+22%) and resistant starch (+115%) was reported by Hazard et al. [23] in the cultivar Kronos, where *SBEIIa* genes were targeted by TILLING.

Subsequently, Hazard et al. [83] pyramiding *SBEIIa* and *SBEIIb* genes found that the mutations in the four starch-branching enzyme II genes of durum wheat resulted in larger increases of amylose and resistant starch content. The presence of the mutations was also associated with an average 5.2% reduction in kernel weight (*p* = 0.0007) and 15% reduction in grain yield (*p* = 0.06) compared with the wild type. Technological quality analysis showed that the mutant lines have acceptable quality, with positive effects on pasta firmness but negative effects on semolina extraction and pasta colour. Positive fermentation responses were detected in rats (*Rattus* spp.) fed with diets incorporating SBEIIa/b-AB pasta compared with controls. The differences included significant increases in cecal contents, decreases in cecal pH and increases in cecal SCFAs. Sissons et al. [84] used semolina obtained from the SSIIa and SBEIIa mutants with high amylose content (43.5% and 57.8%, respectively), to prepare spaghetti, with the objective of reducing the glycaemic index of pasta while maintaining acceptable technological properties. The appearance of the pasta showed that both high-amylose (HA) pastas were darker than Svevo, with the SBEIIa pasta being the darkest but having a more desirable appearance than commercial wholemeal pasta. Both HA pastas had reduced fully cooked times, with SSIIa having the lowest, compared with Svevo pasta, which is very likely due to the reduced amount of starch that can gelatinize in the HA pasta. In addition, the cooked HA pasta was softer, with higher cooking loss but lower stickiness compared with Svevo. The in vitro starch digestion extent decreased in both mutants, but much more in SBEIIa, while the human in vivo GI was only significantly reduced in SBEIIa pasta (50 to 38). Overall pasta quality was acceptable in both mutants, but the SBEIIa mutation provides a clear glycaemic benefit and would be much more appealing than wholemeal spaghetti. In addition, the authors suggested that a minimum RS content in spaghetti of ~7% is needed to lower GI, which corresponded to an amylose content of ~58. High-amylose starches do not gelatinize fully at the typical temperatures used in RVA profiles, resulting in very low pasting viscosities [85].

## 4. Characterization of Soft Durum Wheat

The overexpression of the *Pina* gene was obtained by Li et al. (2014) [86], through a transgenic approach, in order to reduce the hardness of a durum wheat line and observe the effects of the PINA protein on the kernel texture and other characteristics. The analysis of grain hardness showed that the PINA overexpression reduced grain hardness with medium–hard durum wheat grain. Li et al. (2012) [87] also analysed lines coexpressing the HMW-GS 1Ax1 and lines separately expressing 1Ax1 subunit or the PINA protein. Dough mixing analysis of these lines showed that expression of subunit 1Ax1 positively influenced dough strength and overmixing tolerance, while expression of PINA negatively influenced dough resistance to extension. Lines coexpressing 1Ax1 and PINA showed faster hydration of flour during mixing, very likely due to the lower water absorption and damaged starch associated with PINA expression. Moreover, the presence of the 1Ax1 HMW-GS seemed to compensate the negative effect of PINA on dough resistance to extension.

The first commercial durum variety carrying the translocation with the puroindoline genes was Soft Svevo, the soft-textured kernel version of the durum variety Svevo [32]. In a direct comparison of Svevo and Soft Svevo grown at the same location, the single-kernel characterization (SKCS) hardness index of Soft Svevo was ~31, whereas the hardness index of Svevo was ~78.6 [88]. Soft wheat typically averages around 25 for hardness index, hard wheat approximately 75.

When Soft Svevo and Svevo were milled on a traditional hexaploid-type mill along with check varieties of soft white winter and hard red spring wheats, Soft Svevo had a milling profile similar to, and in some cases better than, that of the soft white winter wheat. Svevo had poor break flour and total flour yields (~17 and 52%, respectively), which was to be expected on a hexaploid-type mill [88]. The average flour ash for Soft Svevo was 0.50, compared with 0.62 for Svevo. Furthermore, the starch damage for Soft Svevo was 1.7%, whereas that for Svevo was 6.4% [88]. Although this level of starch damage for Svevo was on a hexaploid-type mill, the levels of starch damage seen in hard-textured durum when milled are prohibitively high for products other than pasta and couscous.

The milling energy of Svevo vs. Soft Svevo was examined for paired, triplicate samples of each from 12 growing locations [89]. Several key parameters were studied, including the energy required to grind wheat (kJ/kg) as well as the energy required to produce 1 kg of flour (kJ/kg), the total flour produced, and the starch damage. At the first break rolls, Soft Svevo required 13.9 kJ/kg, compared with 17.4 kJ/kg in Svevo. Similarly, in the second break rolls, Soft Svevo and Svevo required 19.4 and 29.8 kJ/kg, respectively. The difference in energy requirements was more pronounced when comparing the energy required to produce 1 kg of flour. At the first break rolls, Soft Svevo and Svevo required 153 and 789 kJ/kg flour, respectively. At the second break rolls, Soft Svevo and Svevo required 103 and 288 kJ/kg flour, respectively. These differences amount to between three and five times more energy needed for Svevo compared to Soft Svevo to produce 1 kg of flour. Furthermore, the total flour yield from this milling was 17.7% in Soft Svevo, compared with 8.7% in Svevo. The resultant flour from Soft Svevo had 2.4% starch damage compared with 11.0% in Svevo [89]. Not only was the milling much more efficient for Soft Svevo but produced a higher-quality flour.

Soft Svevo, along with a second soft durum, Soft Alzada, have been examined for baking properties compared to Svevo [90]. Both soft- and hard-wheat products were tested, although continued emphasis was more on bread products. Soft Svevo made sugar snap cookies with a similar diameter to those of the soft white winter wheat Xerpha. However, the soft durum line Soft Alzada made cookies of a similar diameter to the elite soft white wheat on the market [90]. Cookies made by Svevo were quite small, as had been expected for durum. In a similar study using populations made from a cross between Soft Svevo and a number of CIMMYT durum lines, the soft durum populations had excellent soft wheat quality. The sugar snap cookies were as large as or larger than the elite released varieties from the Pacific Northwest [91].

## 5. Introduction of the HMW-GS 1Dx2 + 1Dy12, 1Dx5 + 1Dy10 and (*Gpc-B1*) Allele in Soft Durum

Although soft durum made bread with better quality than the soft white winter wheat Xerpha, the gluten strength and loaf volume were not on par with those of hard wheat [90,92]. One challenge with durum is the lack of the D genome and the HMW glutenin subunits found there. The 5 + 10 and 2 + 12 alleles have a strong influence on gluten properties and strength. Soft Svevo and Soft Alzada showed moderate gluten strength, although comparatively much weaker than that of a hard red spring variety [90]. Three approaches were taken to improve gluten strength of soft durums: introgression into soft durum of the *Glu-D1* alleles of 2 + 12 and 5 + 10 and crossing the functional *Gpc-B1* allele into soft durum, the hypothesis being that introducing the *Gpc-B1* allele would increase protein content and potentially the dough strength.

Two experimental lines, UCRD01-05 and UCRD01-01, which were two 1D-1B chromosomal translocation lines with the 2 + 12 and 5 + 10 alleles, respectively, were crossed with Soft Svevo and carried out to the F5 and F7 generations, respectively [51]. The gluten strength measurements of flour sodium dodecyl sulfate (SDS) and lactic acid solvent retention capacity increased dramatically with the introgression of the 2 + 12 alleles. Furthermore, bread dough mixing qualities improved substantially from a very weak, poor mixing dough without the 2 + 12 alleles to a moderately strong mixing dough resembling that of a hard winter wheat. The introgression of the 2 + 12 alleles also improved the loaf volume by 131 cm^3^, from 762 cm^3^ without 2 + 12, 893 cm^3^ with 2 + 12. Although the 2 + 12 allele, known to be generally weaker, did improve the dough and bread quality substantially, the introgression of the 5 + 10 alleles, known to be stronger, did not have the same effect. The introgression of the 5 + 10 alleles did improve some of the mixing parameters measured with the Mixograph. However, in both the Mixograph and the bread baking, the dough showed too much elasticity and not enough extensibility to create sufficient oven spring. In combination with the other alleles in Soft Svevo, adding the 5 + 10 alleles created a dough that was too strong and not extensible enough [51].

The third approach to improving gluten strength of Soft Svevo was gaining a functional Grain protein content-B1 (*Gpc-B1*) allele in Soft Svevo by crossing with Desert King-High Protein. The protein content of the Soft Svevo with *Gpc-B1* increased by 1.7% along with an increase in the flour SDS sedimentation volume. However, the Mixograph dough parameters were not markedly improved with *Gpc-B1*. Bread loaf volume was similarly improved only marginally [92].

The traditional idea that pasta needed to be made from durum semolina to result in a high-quality product was challenged in a pasta study comparing commercial durum semolina, durum flour and three varieties of soft durum [93]. The pasta weight increase during cooking was similar across the soft durum flour and commercial semolina samples. However, the soft durum samples had lower cooking loss than durum semolina (average 3.93% vs. 5.12%, respectively). Optimum cooking time was also slightly lower in soft durum samples compared with durum semolina. Pasta firmness is an important sensory parameter, contributing greatly to the pleasant pasta-eating experience. Soft durum flour had pasta firmness comparable with that of durum semolina. Additionally, stickiness is a negative attribute in pasta. Soft durum pasta had similar stickiness to, and for some samples, less than that of durum semolina pasta [93].

## 6. Conclusions

Durum wheat has limited uses compared with bread wheat, with pasta being the major end product, and this can be associated with its high hardness and lack of D genome. The use of classical and innovative biotechnological tools has made it possible to modify the processing and nutritional characteristics of durum wheat. In particular, modification of kernel texture and introduction of D-genome HMW-GS have both been achieved via *ph1*-mediated homoeologous recombination and the transfer of genetic material from bread wheat to durum wheat, whereas mutagenesis via TILLING has proved very effective in modifying starch composition.

Durum wheat for pan bread use is limited due to its weak and/or inextensible gluten. The introduction of *Glu-D1* alleles from bread wheat improved dough strength in a range of genetic backgrounds. Although the 1Dx5 + 1Dy10 produced stronger dough than 1Dx2 + 1Dy12, the dough was excessively strong and inelastic, and loaf volume was either inferior or not affected. In contrast, the 1Dx2 + 1Dy12 sometimes improved bread LV particularly when the background genotype had weak gluten strength, as in the Lira biotypes. Generally, greater dough strength did not result in better loaf volume, so the modified durum was still unable to match the bread quality of the hexaploid wheat. It is suggested that dough extensibility needs to be improved to allow higher LV. This could be achieved by exploiting better the genetic variation that exists in durum and/or using the *Glu-D1* gene introgressions.

The use of mutagenesis of the genes involved in starch biosynthesis has permitted selecting durum wheat lines with large variation in amylose content, capable to satisfy the demand of foods with high nutritional value and counteract the onset of important diseases.

Durum wheat kernel hardness has been modified, making milling accessible to those with traditional roller mills without sacrificing pasta quality. Soft durum also has some unique properties that make it a good candidate for niche products like extruded snack foods. The current and future work concerning soft durum and glutenins is focused on lines carrying the 2 + 12 alleles and continuing to make crosses with high-quality soft durum lines for both bread quality and agronomic properties. Soft durum has also shown promise with extrusion for snack foods and breakfast cereals [94]. Currently, there are soft durum lines crossed with waxy (low- to zero-amylose) and high-amylose lines in an effort to create even higher-quality end products with improved characteristics related to the transformation processes and to the consumer demand for healthy food.

## Data Availability

No new data were created or analyzed in this study. Data sharing is not applicable to this article.
